# Dietary practice and associated factors among type 2 diabetic patients: a cross sectional hospital based study, Addis Ababa, Ethiopia

**DOI:** 10.1186/s40064-015-0785-1

**Published:** 2015-01-13

**Authors:** Amelmal Worku, Solomon Mekonnen Abebe, Molla Mesele Wassie

**Affiliations:** Department of Human Nutrition, College of Medicine and Health Sciences, University of Gondar, Gondar, Ethiopia

**Keywords:** Type 2 diabetes, Dietary practice, Yekatit 12 hospital, Addis Ababa, Ethiopia

## Abstract

**Background:**

Dietary management which is considered to be one of the cornerstones of diabetes care is based on the principle of healthy eating in the context of social, cultural and psychological influences on food choice. In Ethiopia, there is lack of data on the dietary practice of diabetic patients which underestimates its role in the management of diabetes. Hence, this study assesses the level of dietary practices and their associated factors among Type 2 diabetic patients in Addis Ababa, Ethiopia.

**Methods:**

Institution-based cross-sectional quantitative study design was employed. A total of 403 study subjects were included in the study. A pretested questionnaire was used to collect data. The collected data were entered into Epi Info version 3.5.3 and exported to SPSS version 20.0 software packages for further statistical analysis. The data were analyzed using bivariate and multivariate logistic regression. The degree of association between dependent and independent variables was assessed using the odds ratio with a 95% confidence interval and variables with p-value ≤0.05 were considered significant.

**Results:**

About 46.4% of the patients were overweight and obese. More than half of the respondents (58.8%) had FBG level ≥ 126 mg/dl. The level of dietary practice among 207 (51.4%) type 2 diabetic patients was poor. Not getting nutrition education in hospitals [AOR = 4.47, 95% CI: (1.92,10.40)], despondency [AOR = 2.15, 95% CI: (1.14,4.02)], facing difficulty to choose foods [AOR = 9.66, 95% CI: (5.12,18.24)], non- availability of fruits and vegetables [AOR = 2.78, 95% CI: (1.03,7.54)], thinking about the high cost of foods [AOR = 2.36,95% CI: (1.18, 4.70)] were the factors significantly associated with the poor dietary practice.

**Conclusion:**

Findings of this study indicated that the majority of the patients had poor dietary practice. Therefore, the integration of diabetic based nutrition education with motivation and home gardening is highly recommended.

## Introduction

Diabetes is one of the rapidly increasing non-communicable diseases and an important public health problem all over the world. Recent estimates from the 2013 International Diabetes Federation [IDF] suggest that the number of adults living with diabetes in the world will rise from 382 million in 2013 to 592 million in less than 25 years (Guariguata et al. 2013).

Sub-Saharan Africa, like the rest of the world, is experiencing an increasing prevalence of diabetes alongside other non-communicable diseases (Hall et al. 2011; Gill et al. 2009). Ethiopia, which is one of the developing nations, is at a risk of increased diabetes incidence. The number of deaths attributed to diabetes reached over 21,000 in 2007. This estimate has increased to about 25,000 in 2011 (Haregu and Alemayehu 2012). Type-2 diabetes constitutes about 85 to 95% of all diabetes in high-income countries and accounts for an even higher percentage in low and middle-income countries (Sicree et al. 2010).

Dietary management is considered to be one of the cornerstones of diabetes care. It is based on the principle of healthy eating in the context of social, cultural and psychological influences on food choices (Ekore et al. 2008). Good diabetes management is a balance between healthy eating, exercise and medication (Control CfD et al. 2011). The problem, however, is that most diabetic patients have difficulty of identifying the recommended quality and quantity of food that they have to eat in order to control their blood glucose level (association SAd: South african diabetes association. In.: South Africa Diabetes Association 2001).

Dietary practice refers to patients’ choices in food consumption based on diabetes nutrition education that gives emphasis to intake of food with lower fat, higher fiber, and lower sodium (Shamsi et al. 2013). A study done in Bahrain indicated that lack of proper professional dietary assessment, follow-up and advice by health care providers were the main influencers on dietary practice of type 2 diabetic patients (Shamsi et al. 2013). Findings from a study in self-care practice and glycemic control among adults with diabetes at Jimma University Specialized Hospital, south-west Ethiopia, showed only 55.6% of the participants had regular meals (Hailu et al. 2012). Another study done at Tikur Anbessa Specialized Hospital, Addis Ababa, Ethiopia, indicated that the majority of the patients had poor adherence to self-care practices, especially in diet management practices (Berhe et al. 2012).

However, in Ethiopia incomplete routine health information and lack of data on the proper dietary practice of diabetic patients affect the long term management of diabetes. These diabetic patients are facing difficulty in choosing food items when they feel like eating. They also fail to decide how much to eat whenever necessary. At the same time their care givers also fail to identify food items to be included in the diabetic meal and how to prepare them. Therefore, this study assesses the level of dietary practice and its associated factors among type 2 diabetic patients in Addis Ababa, Ethiopia.

## Methods

This study was conducted at the Outpatient Department of Yekatit 12 Medical College Hospital, Addis Ababa, Ethiopia. An institution-based cross-sectional quantitative study design was implemented. The study population comprised confirmed persons with diabetes aged 18 years and above attending the Diabetes Referral Clinic. All adult type 2 diabetic patients 18 years and older, visiting the Outpatients Department on daily bases in the months of March to April, 2014, was selected for the study on a daily basis. On the other hand, those who were critically ill and unable to participate in the interview and those who were recently diagnosed (less than one year of diagnosis) were excluded.

Since there was no study done on the dietary practice of type II diabetic patients, we took p value of 50%, with 5% marginal error and 95% CI and a non-response rate of 10%. Based on this assumption, the final sample size of the study was 422, and all eligible patients took part in the study.

### Data collection

Data were collected by interviewing eligible subjects using a pretested structured questionnaire. The questionnaire included questions that assessed dietary practice of patients as dependent variable. Dietary practice was assessed using the modified form of the eight-item Morisky medication adherence scale (MMAS-8) (Morisky and DiMatteo 2011). The dietary practice was assessed using the 11 item scale. Components were computed by taking the mean value to classify the respondents as “good” and “poor”. That is, those who scored below the mean value were classified as Good and those who scored above the mean value as Poor dietary practices. Value 0 was given for good practice and 1 for poor.

The questionnaire included questions that assessed diabetic risk factors, demographic characteristics, wealth status, duration of diabetes, lifestyle, behavioral and social factors, health service related factors, barriers to adherence to dietary practice related factors, and nutritional knowledge. We also gathered information on self-perception by asking how despondency they were with the care they received at the current diabetic clinic (we rated the responses on a two level scale as “Yes” or “No”). In addition, physical measurements were taken using standardized techniques and calibrated equipment. Height was measured using a stadiometer; participants stood in erect posture without shoes and the results were recorded to the nearest 0.5 cm. Measures were taken two times, and the average was considered in the analysis. Body mass index (BMI) was calculated as the ratio of weight in kilograms to the square of height in meters (Grundy et al. 2004). Subjects were weighed to the nearest 0.1 kg in light indoor clothing and bare feet or with stockings. Blood samples were collected from each participant by a trained laboratory technician following aseptic techniques. The blood samples were immediately taken to the hospital laboratory for chemistry analyses. Biochemical tests (FBG) were carried out using 902 Automatic Analyzer with Roche/Hitachi kit. Fasting blood glucose (FBG) was collected early in the morning (after eight hours of fasting) before participants took their breakfast (Grundy et al. 2004). A uniform data abstraction sheet was prepared to gather relevant data (FBG and duration of DM) from the medical records.

An assessment of the dietary practices of the patients was based on the general advice for diabetic diet plan (association SAd: South african diabetes association. In.: South africa diabetes association 2001). An additional structured questionnaire was developed on the bases of variety of literatures (Wen et al. 2004; Collier TL: Dietary Routines and Diabetes: Instrument Development. Ohio University 2007; Yannakoulia 2006; Parmenter and Wardle 1999). The questionnaire was initially prepared in English and then translated into Amharic by the principal investigator and then translated back to English. The Amharic version of the questionnaire was used for data collection.

The data collectors’ team was composed of laboratory technicians, nurses, and supervisors. All were trained by the principal investigator for two days on the procedures of the study. To ensure the quality of the interview and the acquisition of quality data, random checks were carried out by the principal investigator.

### Data quality management

To ensure the quality of the data, training was given to data collectors and the supervisor and a pre-test was also administered to 5% of the total sample size to assess the clarity, length, completeness, and consistency of the questionnaire.

To maintain the quality of the data the supervisor carried out regular supervisions, spot-checking, and reviewing the completed questionnaire. Meanwhile the principal investigator coordinated the overall activity. Training was given to all the data collectors and there was calibration after every measurement.

### Data management and analysis

Data was checked for completeness and cleaned manually. It was then entered using Epi-Info version 3.5.3 and exported to SPSS version 20 for further analysis. A wealth score was computed using the principal component analysis (PCA) form variables which include monthly income, agricultural productivity, household assets, and utility; the assumption of PCA was also checked. Bivariate analysis was used to check the association between independent variables and dietary practice. Logistic regression was applied to test the presence of association. The independent variables (covariates) were selected into the model based on prior evidence in the literature, conceptual framework, and their effect in current analysis.

Independent variables with a p-value of 0.20 and less during the bivariate test were then included in the multivariable logistic regression model to include the marginal confounder. Independent variables in the model were Age, educational status, BMI, wealth, diabetic nutrition education in hospitals, not making food choice, despondency, taking holidays and celebrations as free days to eat, difficulty to choose foods, family and friends support, food planning, availability of fruits and vegetables, thinking about the high cost of foods, nutritional knowledge of the respondents, nutritional status and fasting blood glucose level. Values were then considered statistically significant when p-value is less than 0.05 at 95% CI. Frequency tables, and texts, were used for data presentation.

### Ethical considerations

The ethical approval and clearances were obtained from the Ethical Review Board of the Institute of Public Health, the University of Gondar. Permission letter was obtained from the Yekatit 12 Hospital CEO. Informed verbal consent was obtained from each study participant after the purpose and significance of the study was explained by the data collectors. They were informed of their rights to withdraw from the study at any time.

## Result

### Socio –demographic and economic characteristics

A total of 403 Type 2 diabetic patients have participated in the current study with a response rate of 95.5%. Half of the respondents were males. The mean (±SD) age of respondents was 55.19 (±9.6) years with the minimum age of 30 and maximum of 77 years. Above two-thirds (71.0%) of the participants were Orthodox Christians followed by Muslims (15.1%). More than half (55.1%) of the respondents were married and two hundred twenty-eight (56.6%) of the study population had formal education (Table 1).

**Table 1:** **Socio demographic and economic characteristics of type 2 diabetic patients at Yekatit 12 Medical College Hospital, Addis Ababa, Ethiopia, 2014 (n = 403)**

**Variable**	**Frequency**	**Percent (%)**
**Sex**		
Male	205	50.9
Female	198	49.1
**Age**		
30 - 60	282	70
≥61	121	30
**Marital status**		
Single	49	12.1
Married	222	55.1
Divorced/Separated/Widowed	132	32.8
**Religion**		
Orthodox Christian	286	71
Muslim	61	15.1
Protestant	6	1.5
Others	50	12.4
**Educational status**		
Non formal education	175	43.4
Formal education	228	56.6
**Wealth**		
Poor	117	29
Medium	154	38.2
Rich	132	32.8
**Ethnicity**		
Amhara	191	47.5
Oromo	98	24.3
Guragae	57	14.1
Tigray	44	10.9
Others***	13	3.2

Others***—(Catholic, The 7 days Adventist, Jehovah witness, Wolayita, Hadere).

### Dietary practice

The overall proportion of poor dietary practice among the respondents was 51.4% [95% CI: (46.4, 56.2)]. The proportion with poor dietary practice was 51.23% [95% CI (44.3, 58.0)] among males and 51.52%, [95% CI: (44.5, 58.5)] among female respondents.

The proportion with poor dietary practice was 47.5% [95% CI (41.7, 53.4)] among the age group of 30-60, and 60.3%, [95% CI (51.6, 69.1)] among those who were 61 and above years old (Table 2).

**Table 2:** **Dietary practice of respondents with respect to the eleven variables measuring failure in practice among type 2 diabetic patients in Yekatit 12 Medical College Hospital, Addis Ababa, Ethiopia, n = 403**

**Variable**	**Frequency**	**Percent (%)**
**Forgetting to plan the meals you eat ahead?**		
Yes	168	41.7
No	235	58.3
**Did you miss your dietary plan yesterday?**		
Yes	133	33
No	270	67
**Over the past two weeks, were there any days when you did not take your dietary plan properly?**		
Yes	182	45.2
No	221	54.8
**Do you sometimes forget to comply your dietary plan with everyday life?**		
Yes	151	37.5
No	252	62.5
**When you feel like your DM is under control, do you sometimes stop taking your dietary plan**		
Yes	170	42.2
No	233	57.8
**Do you ever feel hassled about sticking to your dietary plan?**		
Yes	221	54.8
No	182	45.2
**Did you have feelings of dietary deprivation?**		
Yes	299	74.2
No	104	25.8
**Are you rigid, instead of flexible eating to control your DM?**		
Yes	159	39.5
No	244	60.5
**Forgetting to include fruits in your food daily?**		
Yes	157	39
No	246	61
**Do you forget to include vegetables in your food daily?**		
Yes	132	32.8
No	271	67.2
**Do you forget to cut down butter and fat intake in your food?**		
Yes	74	18.4
No	329	81.6

### Health status and available health services

About 65.8% of the respondents had ≥ 5 years duration of diabetic disease. Half of the respondents (49.9%) had other chronic diseases. About 16.2% didn’t get diabetes nutrition education in hospitals (Table 3).

**Table 3:** **Health status and available health services for type 2 diabetic patients in Yekatit 12 Medical College Hospital, Addis Ababa, Ethiopia, n = 403**

**Variable**	**Frequency**	**Percent (%)**
**Duration of the disease**		
<5Years	138	34.2
> = 5Years	265	65.8
**Chronic disease other than DM**		
Yes	201	49.9
No	202	50.1
**Nutrition education in hospitals**		
Yes	337	83.8
No	65	16.2
**I get nutrition journals**		
Yes	129	32
No	274	68
**Visual nutrition education**		
Yes	135	33.5
No	268	66.5

### Barriers to adherence to diet regimen

**Figure 1:**
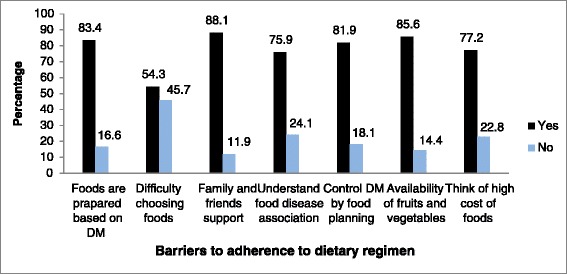
**Barriers to adherence to the dietary regimen of participants of type 2 diabetic patients in Yekatit 12 Medical College Hospital, AA, Ethiopia, 2014.**

**Figure 2:**
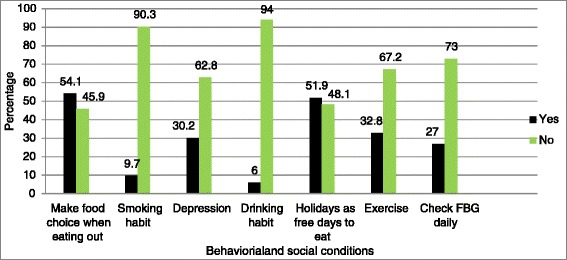
**Behavioral and social conditions of participants of type 2 diabetic patients in Yekatit 12 Medical College Hospital, Addis Ababa, Ethiopia, n = 403.**

Concerning barriers to adherence, 16.6% of the respondents said foods were not prepared based on their disease. Furthermore, 54.3% of the respondents had difficulty choosing foods and 14.4% of the respondents reported non-availability of fruits and vegetables which formed a barrier to adhere and following the diet regimen, (Figure 1). For behavioral and social conditions of participants see Figure 2.

### Nutritional knowledge

Nutritional knowledge was measured by using six variables, number of fruits and vegetables eaten per day, Oils to cut down, Foods to cut down a lot, Identifying Healthy oil, Number of regular meals diabetic patients ate per day, and number of snacks per day. Median was taken to classify the patients as having good or poor nutritional knowledge. Those who scored above the median were classified as having poor nutritional knowledge and those who scored below the median as having good nutritional knowledge.

### Factors affecting the dietary practice of type 2 diabetic patients

The multivariable logistic regression analysis showed that those who did not get diabetic nutrition education were 4.47 times more likely to have poor dietary practice than those who got (AOR = 4.47; 95% CI: 1.92, 10.40). Patients who had despondency were 2.15 times more likely to follow poor dietary practice than those who did not have despondency (AOR = 2.15; 95% CI: 1.14, 4.02). Patients who had difficulty to choose foods were 9.66 times more likely to have a poor practice than patients who didn’t (AOR = 9.66; 95% CI: 5.12, 18.24). Patients who had less access to fruits and vegetables were 2.79 times more likely to have poor dietary practice than those who did not (AOR = 2.79; 95% CI: 1.03, 7.54). Likewise, patients who thought of cost of foods were 2.36 times more likely to have poor dietary practice than those who did not think about cost of foods (AOR = 2.36; 95% CI: 1.18, 4.70), (Table 4).

**Table 4:** **Bivariate and Multiple Logistic Regression Analysis of factors affecting dietary practice of type 2 diabetic patients in Yekatit 12 Medical College Hospital, AA, Ethiopia, 2014**

**Variables**	**Dietary practice**		**COR (95% CI)**	**AOR (95% CI)**
	**Poor**	**Good**		
**Age**				
30 - 60	48	148	1	
≥61	73	134	1.68(1.09,2.58)	
**Wealth**				
Poor	72	45	1.80(1.09,3.00)	
Medium	73	81	1.01(0.64,1.62)	
Rich	62	70	1	
**Get nutrition education in Hospitals**				
No	49	17	3.08(1.76, 5.78)	4.47(1.92, 10.40)
Yes	158	179	1	1
**Despondency**				
Yes	100	50	2.72(1.79,4.16)	2.15(1.14,4.02)
No	107	146	1	1
**Difficulty to choose foods**				
Yes	163	56	9.26(5.87,14.60)	9.66(5.12,18.24)
No	44	140	1	1
**Family and friends support**				
No	37	11	3.66(1.80,7.40)	
Yes	170	185	1	
**Availability of fruits and vegetables**				
No	48	110	5.61(2.75,11.46)	2.79(1.03,7.54)
Yes	159	186	1	1
**Think of high cost of foods**				
Yes	175	136	2.41(1.48,3.92)	2.35(1.18,4.70)
No	32	60	1	1
**Nutritional status(BMI)**				
Underweight	5	3	1.14(0.26,4.92)	
Normal	91	117	0.53(0.36, 0.80)	
**Fasting blood glucose level**				
<126	55	111	1	
≥126	152	85	3.60(2.38,5.48)	

In this study both the bivariate and multivariate analysis showed that there was no statistically significant association between poor dietary practice and religion, marital status, occupation, smoking habit, drinking habit and duration of disease.

## Discussion

In this institution based cross-sectional study, we were able to measure the proportion of poor dietary practice among type 2 DM patients. We found that a large proportion of type 2 DM had poor dietary practice. Not getting nutrition education in hospitals, having despondency, difficulty to choose foods, non-availability of fruits and vegetables and thinking about high cost of foods were the variables identified for having significant associations with poor dietary practice.

The overall occurrence of poor dietary practice among type 2 diabetic patients at Yekatit 12 Medical College Hospital was found to be 51.4%. Studies done on the assessment of dietary practice among diabetic patients in the United Arab Emirates and Riyadh, Saudi Arabia also indicated inadequate dietary practice (Mohamed et al. 2013; Al-Kaabi et al. 2008). Another study done on compliance and control of diabetes in a family practice setting in Saudi Arabia has indicated that there was a 60% poor diet compliance which is higher than the finding present study. The disparity could be explained by the variation in the settings of the study, difference in socioeconomic status, as well as difference in the types of foods available in the two countries (Khattab et al. 1999).

Not getting diabetic nutrition education at hospitals was one of the main factors that were identified to have association with the poor dietary practice of the patients (Tan et al. 2011). This is in line with a report from South Africa which has identified the need for nutrition education related to diabetes care for optimal diabetes management (Hjelm and Mufunda 2010). This may be due to the fact that those who get nutrition education follow the advices from clinicians and have better knowledge and understanding about the food-disease association, food guides and prescriptions than those who don’t get nutrition education.

Despondency was another factor identified for poor dietary practice. This result is in agreement with a report that showed coexisting despondency in people with diabetes is associated with decreased adherence to treatment, poor metabolic control, and decreased quality of life (Egede and Ellis 2010). Accordingly, those who were despondency for most of the times were twice highly at risk of forgetting and not giving value to food planning and therefore consume whatever is edible.

Difficulty to choose foods was also identified as a factor for poor dietary practice. This may be due to cultural and personal food choice, economic reasons, the unavailability of food guide prepared for diabetic patients in the country and lack detailed understanding of the food-disease association.

Non availability of fruits and vegetables was another factor affecting the dietary practice of diabetic patients. This result is in line with a report on Creating Healthy Food and Eating Environments in the United States of America (Story et al. 2008). This may be due to the seasonality of fruits and vegetables which make the patients suffer from difficulty to take the recommended type and amount of fruits and vegetables, leading to poor dietary practice.

Respondents who thought about the high cost of foods were over 2 times more likely to have poor dietary practice than those who did not think about the high cost of foods. This result is in agreement with a study done on Iranian adults with diabetes which showed thinking about cost of foods as the most frequent barrier to the dietary practice among type 2 diabetic patients (Jazayeri and Pipelzadeh 2006). This commonly corresponds to the economic background. In clear terms, those who have economic constraints cannot have enough money to buy different types of foods to fulfill their daily requirements. Therefore, they will be forced to consume only some specific foods without choice and get exposed to poor self dietary management.

This study may not show temporal relationships of potential risk factors with dietary practice due to cross sectional nature of the design used. Using self-reported dietary practice as a measure of the level of practice may introduce social desirability bias. The dietary practice scale has not been validated before, and it is likely that our estimates may underestimate or overestimated the outcome. The study will be the base line for future studies since there is limited evidence on the dietary practice of diabetic patients in Ethiopia.

## Conclusion

The prevalence of poor dietary practice was observed in more than fifty percent of the patients; it is therefore a major public health problem. Not getting diabetic nutrition education at hospitals, being despondency, difficulty of choosing relevant foods for their specific health problems, non-availability of fruits and vegetables, and thinking about the high cost of foods were important factors affecting dietary practices of type 2 diabetic patients. Finally, we would like to recommend to the health practitioners to give more attention to diabetic patients’ nutrition education programs in hospitals. It is necessary to prepare diabetic nutrition guidelines at a national level. Improving production of fruits and vegetables by the agricultural sector and increasing market availability of these food items is highly recommended in this study.

Health care services should also empower patients to heal themselves by addressing the causes of their disease and facilitating lifestyle changes through health promotion.
